# A necessary considering factor for breeding: growth-defense tradeoff in plants

**DOI:** 10.1007/s44154-023-00086-1

**Published:** 2023-04-06

**Authors:** Hong Zhang, Yuanming Liu, Xiangyu Zhang, Wanquan Ji, Zhensheng Kang

**Affiliations:** 1 State Key Laboratory of Crop Stress Biology for Arid Areas, College of Agronomy, Northwest A&F University, Yangling, Shaanxi 712100 People’s Republic of China; 2 State Key Laboratory of Crop Stress Biology for Arid Areas, College of Plant Protection, Northwest A&F University, Yangling, Shaanxi 712100 People’s Republic of China

**Keywords:** Crops, Growth-defense, Reasonable cell death, Yield penalty

## Abstract

Crop diseases cause enormous yield losses and threaten global food security. Deployment of resistant cultivars can effectively control the disease and to minimize crop losses. However, high level of genetic immunity to disease was often accompanied by an undesired reduction in crop growth and yield. Recently, literatures have been rapidly emerged in understanding the mechanism of disease resistance and development genes in crop plants. To determine how and why the costs and the likely benefit of resistance genes caused in crop varieties, we re-summarized the present knowledge about the crosstalk between plant development and disease resistance caused by those genes that function as plasma membrane residents, MAPK cassette, nuclear envelope (NE) channels components and pleiotropic regulators. Considering the growth-defense tradeoffs on the basis of current advances, finally, we try to understand and suggest that a reasonable balancing strategies based on the interplay between immunity with growth should be considered to enhance immunity capacity without yield penalty in future crop breeding.

## Introduction

Plants are crucial food sources for humans and animals. To ensure global food security, further increases by about 70% in food production are expected for the estimating 9.7 billion world population in 2050 (United Nations DoEaSA, Population Division 2022). In the last decades, a huge of increases in crop yields were achieved due to ‘Green Revolution’ and new breeding methods utilization, including marker-assisted selection (MAS), transgenes and gene-editing (Litrico and Violle 2015; Uauy et al. 2017). During development and reproduction, plant could only alter intrinsic gene expression or protein modification rather than eluding from parasite invasion to improve performance under adverse environment. In agriculture, to combat widespread microbial pathogens, and minimize major losses in yield, the use of resistance (R) genes is thought as an economical, environmentally safe method to control plant diseases in crop breeding programs. Plants wield two classes of immune resistance, pathogen-associated molecular pattern (PAMP)-triggered immunity (PTI) and effector-triggered immunity (ETI), to prevent further pathogen progress (Jones et al. 2016; Wilkinson et al. 2019). The hypersensitive response (HR) is superimposed upon the basal resistance and triggered by R genes, characterized by a rapid death of plant cells at and around infection sites by pathogens, such as bacteria, fungi, and viruses (Valandro et al. 2020). HR is a specialized form of regulated cell death (CD) that occurs specifically under stress conditions (Balint-Kurti 2019; Locato and De Gara 2018), which destined to impair plants’ development and reduce final yield in the way of more or less. Additionally, a large number of empirical breeding practice examples the fact that the lines with high levels of resistance usually carry yield penalties, while high-yield lines intuitively have a weak resistance level to pathogen. This was also substantiated by several reports, especially by wild-relative species segments introgressed lines, which are potential bridge materials in future breeding. For example, the wheat (*Triticum aestivum*) streak mosaic virus R gene *Wsm1* (transferred from *Thinopyrum intermedium*) and stem rust R gene *Sr26* from *Agropyron elongatum* are associated with a mean yield reduction of 21% and 9%, respectively (Brown 2002), as well as the barley (*Hordeum vulgare*) *mlo* has a 5.2% TGW (thousand-grain-weight) reduce and 4.2% yield penalty (Ning et al. 2017). Two recently reported substitution line, wheat-Ph1D (*Psathyrostachys huashanica*) and wheat–Th1J^s^(1D) (*Thinopyrum ponticum*) confer resistance to powdery mildew and stripe rust in wheat but causing a significant spike length shorten and tiller inhibition (Qu et al. 2022; Wang et al. 2020d). Taken the possibility that a single gene can be linked to susceptibility to one fungal species but related to resistance to another species (Howard et al. 2010), these hint that it is only an idealized view to obtain lines with both excellent high-yield agronomic traits and ideal resistance to pathogens in crop empirical breeding program. Hence crops breeders have to balance the disease resistance and high-yield traits during selection from the breeding populations. However, the forming reason or mechanism was not well illustrated because of the limitation of experiments on the effect of disease resistance on crop performance under pathogen evading. Hence scientists have tried to understand growth-defense trade-offs (He et al. 2022; Huot et al. 2014; Karasov et al. 2017; Ning et al. 2017). Here, we summarized present literatures trying to illuminate the tradeoffs between development and disease defense in crop plant with a distinguishing perspective, and thereby focused on how crop breeder reasonably balance the costs of resistance in crops and the likely benefits of resistance genes in new cultivars combining other aspects of the plant’s phenotypes, especially in maximizing yield-related agronomy traits.

### Development and immunity trade-off in initiating signal system

In plants, cell death (CD) occurs during development, defense response or when exposed to adverse conditions. It is no doubt that CD, especially in programmed cell death (PCD) and/or regulated cell death (RCD), is happened at a fitness cost of energy, which sometimes is associated with yield costs’ (Nejat and Mantri 2017; Nelson et al. 2018; Ning et al. 2017). At the host–pathogen interface, the protein composition of the plasma membrane (PM) has important implications for how a plant cell perceives and responds to invaders (Ekanayake et al. 2019; Jones et al. 2016; Viotti et al. 2010). An important process was to add or remove cargo proteins (such as pattern-recognition receptors, transporters, and other proteins with immune functions) to or from the PM via membrane-bound vesicles in plant immune responses (LaMontagne and Heese 2017; Viotti et al. 2010), which further activates the signal for downstream responses. The ability to modulate its PM composition is critical for regulating the strength, duration, and balance of immune responses with necessary development in animal and plant (Eich et al. 2016; Man et al. 2021; Offringa and Huang 2013). In addition, plant cell unlike to animal, in which cell wall (CW) is also a highly dynamic structure component that is constantly remodeled during growth and development for cell division, elongation and response to environmental cues, including pathogen infection (Hematy et al. 2009).

**Fig. 1:**
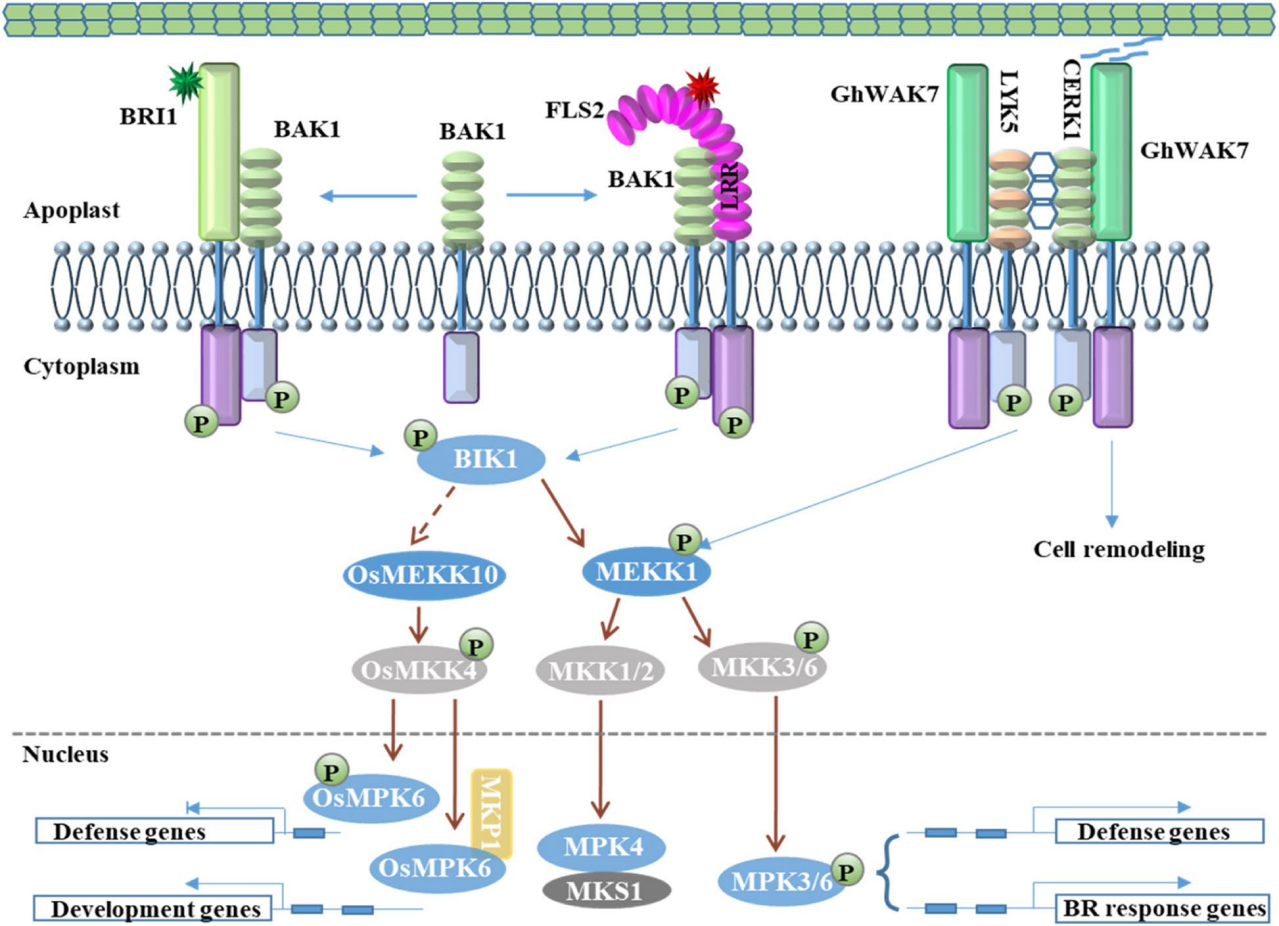
Schematic illustrations SERKs, WAKs and MAPK cassette trade-off in regulating development and defense gene expression. The cell wall is indicated in a format with green brick bars. Note that plasma membrane components interact with each other in a competed manner to display with left and right arrows. The outline and shapes of RLK structures tethering PM are purely schematic; their size and number are only simulating protein domains. The signal P represents phosphorylation in different components and the phosphorylated order is schematic with arrows flow

For colonization, pathogens have to firstly germinate and penetrate waxy cuticular “skin” layer or successfully congest anti-microbial compounds through secreting a battery of cell wall-degrading enzymes (Hematy et al. 2009; Wan et al. 2021). CW is a paramount plant’s structure that fences and protects the plant cell, and it has also emerged as a component of important systems that monitor cell expansion, plant growth and defense (Bacete et al. 2018; Wolf 2017). In which, cell wall-associated kinases (WAKs), an important subgroup of receptor-like kinases (RLKs), link the plasma membrane to the carbohydrate matrix (Kohorn and Kohorn 2012). Similarly, another class of important RLKs (e.g. FLS2, EFR, CRK10, CERK1, LYK5 and BRI) or receptor-like proteins (RLPs) reside in plasma membrane, which are developed for maintaining the cell wall integrity (CWI) and detecting the change in environment and/or hormone levels. In unstimulated plants, botrytis-induced kinase1 (BIK1), a receptor-like cytoplasmic kinase (RLCK), strongly interacts with FLS2, EFR, CERK1 (Zhang et al. 2010). However, upon binding to the bacterial flagellin peptide flg22 (Chinchilla et al. 2007), FLS2 rapidly associates with another RLK, BAK1/SERK3 (BRI1-associated receptor kinase 1), to activate defenses (Sun et al. 2013b). Intriguingly, BRI1 (Brassinosteroid Insensitive 1) also interacts with BAK1 in the presence of brassinosteroids (BRs) and to active BR signal regulating plant development (Choudhary et al. 2012; Sun et al. 2013a). Meanwhile, wheat TaBRI1 interacts with somatic embryogenesis receptor kinase (TaSERKs) leading to the early flowering, increased silique size and seed yield (Singh et al. 2016) in Arabidopsis, although whether TaBRI1 is involved in development and/or immunity in wheat is still unknown. Cell wall-associated kinases (WAKs) are usually linked to the pectin fraction of the cell wall serving as receptors for cell expansion, in which mPectin monitoring activates BR signaling (Yang 2022). However, they are also amenable to short oligogalacturonic acid fragments (OGs) generated during wounding or pathogen exposure though damage-associated molecular patterns (DAMP) (Kohorn and Kohorn 2012; Stephens et al. 2022). Evidence is emerging that WAKs were implicated into the pathogen defense (Guo et al. 2021; Harkenrider et al. 2016; Li et al. 2009; Zhang et al. 2020). The chitin-induced GhLYK5-GhCERK1 dimerization and phosphorylation contributes to cotton defense against *Verticillium dahliae* (*Vd*) and *Fusarium oxysporum* f. sp *vasinfectum* (*Fov*) though interacting with GhWAK7A (Wang et al. 2020a). GhWAK7 belongs to ‘RD’ WAK characterizing with a conserved arginine (R) immediately preceding the aspartate (D) in the catalytic loop of the kinase domain. Notably, RD kinases are more commonly linked to roles in growth and development (Stephens et al. 2022). Analyzing EFR-WAK1 chimeras evidenced that the WAK1 ectodomain is capable of activating the EFR kinase domain upon stimulation with OGs, while the EFR ectodomain could also activates the WAK1 kinase upon stimulation with the cognate ligand elf18 (Brutus et al. 2010). Additionally, WAK signaling responses to pectin in the case of *WAK2* regulating both cell expansion and defense response in model plant accompanied by the induction of *MPK3* (Kohorn et al. 2009, 2012). In wheat, *TaWAK32b*, a homologous gene to *WAK2*, responds positively not only in resistance to powdery mildew stress but also shows differently expression in large grain compared with small grain spikes (Wang et al. 2023; Zhang et al. 2021a). This demonstrated that a crosstalk and/or competition existed in the cell wall signaling (CWS) system between immunity and development due to the balance competition between longer HG fragments and OGs (Choudhary et al. 2012; Lozano-Duran and Zipfel 2015; Wolf 2022). Apart from aforementioned crosstalk, a recently example give a new evidence of protein functional multiplicity due to alternative phosphorylation in growth and defense. The plasma membrane intrinsic proteins (PIPs) carry extracellular domains that are mandatory for perceiving external signals, inducing various responses in plants. TaPIP2;10 relies on phosphorylation at the serine residue S280 to support wheat photosynthesis and productivity, however it confers innate immunity against pathogens through phosphorylation at S121 (Lu et al. 2022). Thus, immunity signaling must be precisely regulated to ensure that in the absence of pathogen infection, the plant allocates resources to growth (Fig. 1). Meanwhile, plants undergoing pathogen attack must integrate multiple signaling pathways and reallocate resources to against infection, as well as guarantee growth to maximize grain size.

### Plant immunity and growth pathways are intertwined and antagonistic on MAPK cassette

To adapt various environmental challenges, plants need to balance diverse signal inputs which involves crosstalk between different signaling pathways. A well-known strategy for integrating these signals is through mitogen-activated protein kinase (MAPK) cascades that play essential roles in signal transduction and amplification for many distinct cellular responses (Jiang et al. 2018). MAPK cascade modules are conserved in all eukaryotes and consist of three kinase components: MAPK kinase kinases (MAPKKKs) phosphorylate dual-specificity MAPK kinases (MAPKKs), which then phosphorylate and activate the terminal MAPKs (Taj et al. 2010). To combat pathogen infection, the AtWRKY33 protein interact with MPK4 indirectly to regulate the expression of defense gene *PAD3* (*phytoalexin deficient 3*) by the activated MEKK1–MKK1/2–MPK4 module after bacterial pathogen attack (Qiu et al. 2008), while MPK3/MPK6 could direct phosphorylate AtWRKY25/33 in PAD3 regulation (Mao et al. 2011). Phenotypic analyses showed that *OsWRKY53* overexpression led to increased grain size as an importantly positive regulator of rice BR signaling after phosphorylation by the OsMKK4-OsMPK6 cascade (Liu et al. 2015a; Tian et al. 2017), while OsWRKY53 functions as a negative feedback modulator of MPK3/MPK6 and thereby acts as an early suppressor of induced defenses though interacting with OsMPK3/OsMPK6 (Hu et al. 2015). The *grain size and number1* (*GSN1*) encodes the mitogen-activated protein kinase phosphatase OsMKP1, and directly interacts with and inactivates the mitogen-activated protein kinase OsMPK6 to regulate the role of OsMKKK10-OsMKK4-OsMPK6 cascade in determining panicle architecture and grain size (Guo et al. 2018; Xu et al. 2018). Whereas, OsMPK6 negatively regulates rice disease resistance to bacterial blight (Yuan et al. 2007), while null mutation of MKP1 in Arabidopsis resulted in growth defects and constitutive defense responses to *Pseudomonas syringae* (Bartels et al. 2009). Another example for MPK6 balancing grain development and resistance to *Xanthomonas oryzae* pv. *oryzae* is to interact with OsVQ13 activating OsWRKY45-dependent JA pathway (Uji et al. 2019). Similarly, Overexpression of *OrMKK3* influenced the expression levels of the grain size-related genes *SMG1*, *GW8*, *GL3*, *GW2*, and *DEP3* functioning morphology and grain size (Pan et al. 2021). BR‐signaling kinase 1 (BSK1) and receptor‐like cytoplasmic kinases (RLCK) group VII members directly phosphorylate MEKK3/5 and activate MAPK cascade followed by the up‐regulation of defense genes expression (Wang et al. 2020b). As shown in Fig. 1, the MAPK cascade is shared by morphogical establishment of many traits, which hinted that this critical joint must be competed when plant face pathogen infection in an important stage of plant development. This means that understanding of MAPKs’ regulation mechanism to provide both durable resistance against pathogens and other agronomic will undoubtedly benefit the development of future crop varieties.

### Immunity and development battle for nucleocytoplasmic transport though nuclear envelope channel

**Fig. 2:**
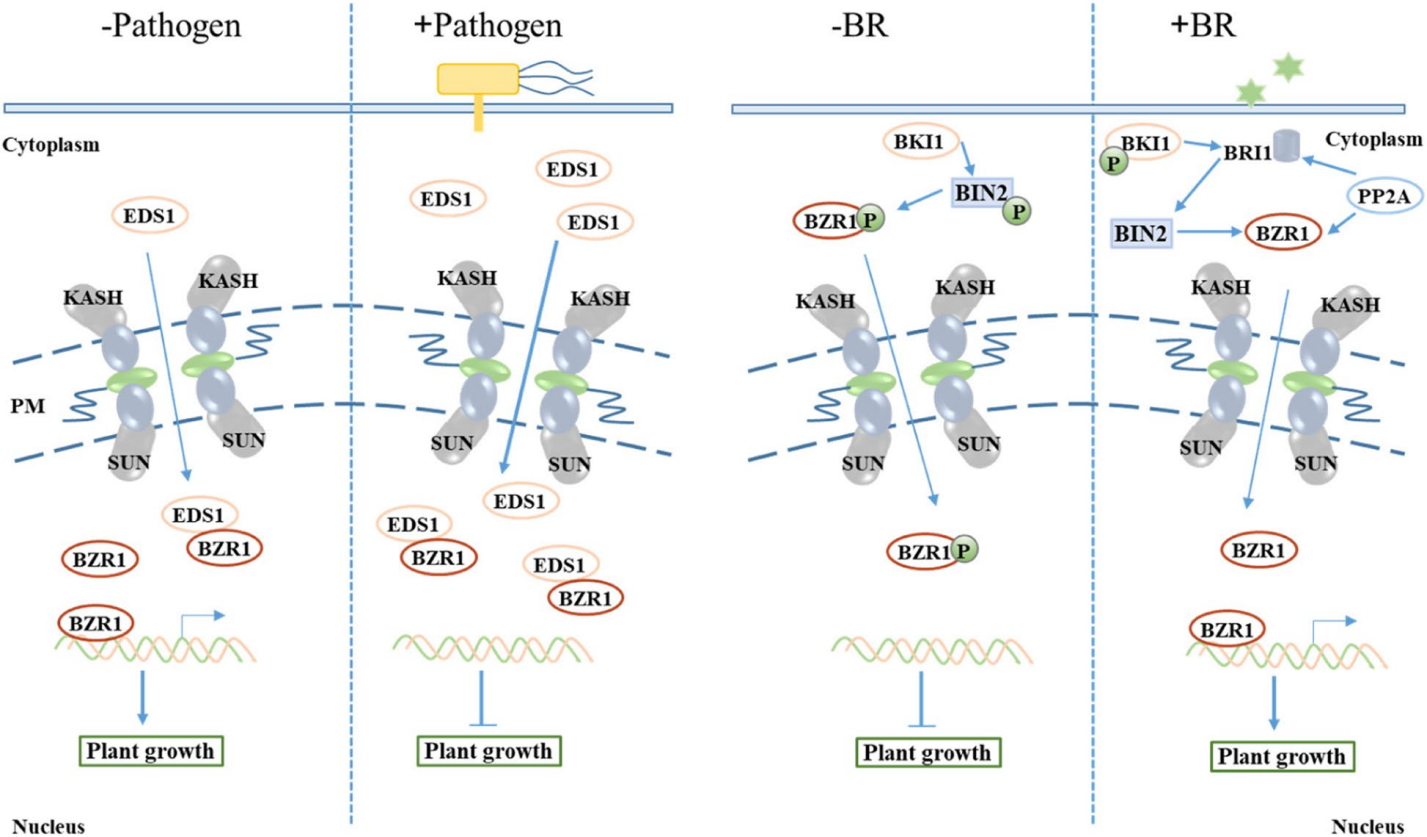
Diagram depicting nucleocytoplasmic transport and transcription factor BZR1 coordination tradeoffs in regulating development and defense gene expression. The cell wall is indicated in a simplified format with blue bar. Note that nuclear envelope channel components interact with each other in manners that are too complex and manifold to detailed display. The effector classes were given on the top based on the stimulated factors. The regulation mechanism of NPC in functional genes was simulated in the middle and bottom panel, including nucleocytoplasmic shuttle and competitor/decoy (transcript factors (TFs) binding targets/protein inhibitors or phosphorylation inactivation). The coiled double lines represent the regulated gene by TFs, like BZR1, and the latter could be dephosphorylated by PP2A. The outline and shapes of NPC components and the tethered regulators are purely schematic; their size have no meaning. The regulated genes could be any potentially functional gene, including development-related genes, R genes and their regulator (e.g. transcription and splicing factors)

Nuclear pore complexes (NPC), also known as SUN–KASH (INM Sad1/UNC-84 and Klarsicht/ANC-1/Syne-1 homology) nuclear envelope (NE) bridges are responsible for connecting the nucleoplasm and cytoplasm in eukaryotes (Graumann and Evans 2017; Yang et al. 2017; Zhou et al. 2012, 2015b). Nuclear transport of immune receptors, signal transducers and transcription factors is an essential regulatory mechanism for immune activation (Gu et al. 2016). Ras-related nuclear protein (Ran) GTPases are required for nucleocytoplasmic transport, mitotic progression, and nuclear envelope assembly in plants (Choudhury et al. 2021; Ma et al. 2007). A WPP domain-interacting proteins (AtWIP) were identified as plant specific KASH proteins, while Ran GTPase activating protein 1 (RanGAP1) is also a member of the WPP domain-containing protein family (Jeong et al. 2005), which is associated with the NE by protein–protein interactions (Xu et al. 2007). Intriguingly, RanGAP2 was identified as a potato virus X resistance protein Rx-interacting protein (Sacco et al. 2007), while TaWPP1 was substantiated to interact with Recognition of *Peronospora parasitica* 13-like protein (RPP13L1) enhancing resistance to powdery mildew in wheat (Zhang et al. 2022b). Guanine nucleotide exchange factors (GEFs) and GTPase accelerator proteins (GAPs) are the switch between GTP-bound active form and GDP-bound inactive form GTPase (Feher and Lajko 2015; Ridley 2006), including the small Rho (Ras homolog) of plants (ROP) type and Ran type GTP-binding (or G-) proteins in plant. Overexpression of *AtRan1* in Arabidopsis leads to promoted elongation growth and enhanced disease resistance against *P. syringae* DC3000 (Xu et al. 2021). Under biotic and abiotic stresses, *TaRAN1* expression was largely unaltered, while *TaRAN2* showed stress specific response (Choudhury et al. 2021). In addition, upon activation by immunoreceptors, NE protein constitutive expresser of PR genes 5 (CPR5) undergoes an oligomer to monomer, and releases cyclin-dependent kinase inhibitor (CKI) for ETI signaling and reconfigures the selective barrier to allow significant influx of nuclear signaling cargos through NPC (Gu et al. 2016; Wang et al. 2014a). Similarly, the AtSUN–AtTIK complexes are substantiated involving in root development (Graumann et al. 2014; Zhou et al. 2015a). Whatever, for defense and/or development, plants have to transfer the related cargoes, corresponding gene and proteins, though the NE bridges, and thereby destined a traffic jam and competition (Fig. 2). An important example is transferring of transcription factors (TFs), which are the most important regulators that control genes expression though binding the upstream specific nucleic sequence motif of functional genes (Spitz and Furlong 2012). Since TF proteins was synthesized in cytoplasm but function in the nuclei, the phosphorylated and/or dephosphorylated TFs are selectively translocated into the nucleus upon mechanotransduction force-activation (Kassianidou et al. 2019; Tapley and Starr 2013). In addition, knocking out nucleoporin (NUP) 85-like protein-encoded genes rescues the cell-death phenotype in double mutant *bak1-3 bkk1-1*, which suggested nucleocytoplasmic trafficking is essential for BAK1- and BKK1-mediated cell-death control (Du et al. 2016). Since *BAK1* and *BKK1* were also involved into the BR regulated signal (Choudhary et al. 2012; He et al. 2007), we inferred that *NUP85* should play a role in plant development as well. Although the grain size related mechanism of NE complex factors is real limitation, we think more and more evidences will be found using fluorescence-based quantification of nucleocytoplasmic transport mothed (Kelley and Paschal 2019).

### Pleiotropic functional genes affect the balance between immunity and grain size

*Brassinazole-resistant* (*BZR*)*,* a key bHLH-type transcription factor (TF) in BR signaling, plays dual roles in plant development and defense. Overexpression of *ZmBES1*/*BZR1-5* in Arabidopsis and rice both significantly increased seed size and weight (Sun et al. 2021), while *TaBZR2* confers broad-spectrum resistance to the stripe rust fungus by activating Cht20.2 transcriptions in wheat (Bai et al. 2021). EDS1 interacts with BZR1 and suppresses its transcriptional activities, and cytoplasmic BZR1 facilitated AvrRps4-triggered dissociation of EDS1 and RPS4 by binding to EDS1, thus leading to RPS4-controlled ETI activation (Qi et al. 2021). Notably, when plants are growing rapidly, the BZR1-mediated suppression of immunity (Lozano-Duran et al. 2013), suggesting that BZR1 acts as an important regulator mediating the trade-off between growth and immunity upon integration of environmental cues (Fig. 2). Similarly, WRKY45 is required for *Panicle blast 1* (*Pb1*)-mediated rice blast resistance (Inoue et al. 2013), and also associates with Pi36, Pib, Pit, Pita, and Piz-t (Liu et al. 2015b), suggesting that WRKY45 may functions as a common module of rice ETI. However, both overexpression of WRKY45 and pyramiding of the *Pi* genes leads to defects in plant growth and result in yield loss (Goto et al. 2015; Peng et al. 2021). Additionally, staurosporine and temperature sensitive 3 (STT3) is a catalytic subunit of oligosaccharyltransferase. In Arabidopsis, BAK1/SERK3, BKK1/SERK4, and BAK1-interacting receptor-like kinase 1 (BIR1) negatively regulate the process of cell death (Gao et al. 2009), and STT3a was identified as an important regulator of bak1/serk4 mutant-associated spontaneous cell death in seedling (de Oliveira et al. 2016). In wheat, *TaSTT3a* and *TaSTT3b* were identified as the functional genes in both defense against *R. cerealis* and increasing grain weight (Zhu et al. 2022). The editing of susceptible gene *mlo* confers powdery mildew resistance in wheat, but the development of the *mlo*-eidted plants were also affected resulting in yield penalty (Wang et al. 2014b). The nonexpressor of PR genes 1 (NPR1) protein has been found to be a key transcriptional regulator in some plant defense responses (Sugano et al. 2010; Wang et al. 2020c). It not only plays an essential role in salicylic acid (SA)-mediated systemic acquired resistance and rhizobacterium-triggered induced systemic resistance, but also is involved in crosstalk inhibition of jasmonic acid (JA)-mediated defense responses (Dong 2004). Blast (caused by *Magnaporthe oryzae*) resistance tests using *OsNPR1* knockdown and overexpressing rice lines demonstrated the essential role of *OsNPR1* in benzothiadiazole (BTH)-induced blast resistance (Sugano et al. 2010). Unfortunately, the OsNPR1-overexpressed lines also displayed phenotypes with decreases in root system, seed number and weight, internode elongation, and tiller number (Li et al. 2016). Another direct example is the hybrid necrosis *Ne2* gene, which encodes NB-LRR resistance gene *Lr13* to leaf rust but hampers the growth of plant exhibiting gradually premature senescence or even death when cooperating with *Ne1* (Hewitt et al. 2021; Zhang et al. 2022a).

The ideal plant architecture seems to be an ideal idea for develop super crop varieties. Ideal Plant Architecture 1 (IPA1) encodes a transcript factor *OsSPL14* and involved into rice growth and development binding to the promoter of *DEP1*, *WRKY51* and *WRKY71* (He et al. 2021; Wang et al. 2018). The higher expression levels of *IPA1* enhance immunity to *M. oryzae* through Ser 163 phosphorylation activating WRKY45 expression after pathogen attack, as well as increases grains per panicle and plant height in a case of dephosphorylating S163 (Wang et al. 2018), but retards seed germination and early seedling growth because negatively regulates starch metabolism (He et al. 2021). Broad-Spectrum Resistance-Digu 1 (Bsr-d1) binds to the promoter of the peroxidase gene *Os10g39170* and *Perox3*, which are involved in basal resistance to rice blast (Li et al. 2019; Zhu et al. 2020). The bsr-d1 mutant confers non-race-specific resistance to *M. oryzae* because of the reduced gene expression of *BSR-D1* (Li et al. 2017), and also affects amino acid and unsaturated fatty acid (UFA) metabolism (Zhu et al. 2020). Plant unsaturated fatty acids plays multiple roles in responding to various biotic and abiotic stresses (He and Ding 2020). The nucleotide-binding leucine-rich repeat (NLR) receptor *PigmR* is constitutively expressed and confers broad-spectrum rice blast resistance but has a negative effect on grain yield. *PigmS*, a *PigmR*-linked gene loci, is highly expressed in pollen and panicles contributing a relative increase in grain weight but dampens *PigmR*’s effect on blast resistance (Karasov et al. 2017). Similarly, the recessive proline-rich protein *pi21* confers resistance to a broad-spectrum of *M.oryzae* but links to an undesirable gene conferring poor flavor and grain quality (Fukuoka et al. 2009). This unexpected genetic link will hamper the utilization of disease resistance genes in crop improvement.

Taken these aforementioned results together, it is not difficult to infer that pleiotropic functional genes, including TFs and other functional genes, could also affect the balance of plant immunity and development.

### How plant reasonably control cell death to help itself balancing fungal immunity and yield

In a breeding program, many agronomy factors must be weighed against one another, and disease resistance is usually not thought as the most important one. Whereas resistance to one or several diseases must be considered when deciding whether or not to market a cultivar in a certain agro-ecological production zone with perennial disease endemic. Large number of empirical breeding practices, no published written or oral histories attest that the successful leading cultivars commonly have a balance capacity in disease resistance and agronomic traits, i.e. overexpression of a molecule with defense activity should be balanced with potential yield during selection from breeding program. Genome-editing was thought as a powerful tool to improve crop resistance without yield penalty (Li et al. 2022). However, the identification of susceptible genes (Wang et al. 2022) is more infrequent and difficult than resistance genes using standard genetic studies because the observing limitation of phenotyping segregation in breeding practice, especially in polyploidy crop species. Additionally, most resistance genes showed specific characterization, meaning that one R gene resistance to an avirulence race but susceptible to another virulent race, and vice versa (Kang et al. 2020), while a single gene can be linked to susceptibility to one fungal species but related to resistance in another fungi (Howard et al. 2010; Zhang et al. 2022b). For example, a plant that has been rationally primed for resistance to biotrophic pathogens may not be able to efficiently respond to necrotrophs due to trade-offs between salicylic acid (SA) and jasmonic acid (JA) pathways (Karasov et al. 2017). Given many aforementioned intersections of immunity and development play a positive or negative role in the trade-off between immunity and plant growth, the expression of some joint genes in plants should be optimum to produce reasonable and opting resistance with minimal effects on plant growth and yield (Huot et al. 2014). The primary challenge is to breed high yield varieties with effective, stable and broad-spectrum resistance. Therefore, the utilization of partial, quantitative and/or adult-plant resistance genes with possible race non-specificity HR was thought as a reasonable way to develop elite varieties (He et al. 2011; Nelson et al. 2018), in which we would like to replenish a primary condition that is balancing yield penalty and disease damage risk. We know that not all photosynthesis energy can be used for producing crop seeds (Parry et al. 2011; Simkin et al. 2020). Hence, partial loss or reduction of photosynthetic ability will not reduce the grain yield of crop plant. Spotted leaf or lesion mimic genes commonly trigger the cell death phenotype appeared after all the leaves stretched in the adult plant (Fan et al. 2018; Tang et al. 2013; Zhang et al. 2021b), which have little influence in the crop yield comparing with non-symptom plants. Conversely, lesion mimic could enhance board-spectrum, adult plant resistance to multi-fungal pathogen, and thereby it maybe more safe than green variety due to balance immunity and development. In addition, *PigmR* confers broad-spectrum resistance in rice with yield penalty, while *PigmS* is associated with a relative increase in grain yield but attenuates resistance. This means that *PigmS* and *PigmR* could be used to balance high disease resistance and yield through epigenetic regulation of paired antagonistic in hybrid varieties as a tool to develop elite crop varieties (Deng et al. 2017). Since the bsr-d1 mutant does not cause obvious yield penalties, utilization of BSR-D1 alleles in rice germplasm might provide a new approach for selecting resistant rice cultivars. These supplied precious resources and cues for us to understand growth–defense tradeoffs occurred in plants, which demand prioritization towards either growth or defense, depending on external and internal factors.

## Conclusions

A breeder has to consider multiple factors during developing a cultivar. By contrast, in the academic world, different scientist study agronomic properties and disease; and most often, different people study different diseases or at least different classes of disease. Therefore, comprehensive considering pathogen defense and crop yield will help us to understand how and what we should do to improve the crop varieties’ performance facing the global food security problem underlining the climate change. Although directly key experiments on the effect of disease resistance on crop performance in the absence of disease is limitation, a breeder’s most effective strategy may not be to select for excellent resistance (if that means sacrificing yield or quality), but to select for reasonable moderate resistance lines with excellent agronomy performances. It’s time to put the pieces back together and to study how to balance responses to disease and crop yield as an integrated part of the crop plant biology.

## Data Availability

Not applicable.

## Abbreviations

PAMP: Pathogen-associated molecular pattern
PTI: PAMP-triggered immunity
PCD: Programmed cell death
HR: Hypersensitive response
CWI: Cell wall integrity
RLK: Receptor-like kinase
RLCK: Receptor‐like cytoplasmic kinase
BIR: BAK1-interacting receptor-like kinase
MAPK: Mitogen-activated protein kinase
NPC: Nuclear pore complex
RanGAP: Ran GTPase activating protein
PIP: Plasma membrane intrinsic protein
CKI: Cyclin-dependent kinase inhibitor
BZR: Brassinazole-resistant
DAMP: Damage-associated molecular Pattern
ETI: Effector-triggered immunity
RCD: Regulated cell death
CWS: Cell wall signaling
WAK: Cell wall-associated kinase
RLP: Receptor-like protein
SERK: Somatic embryogenesis receptor kinase
PM: Plasma membrane
NE: Nuclear envelope
Ran: Ras-related nuclear protein
WIP: WPP domain-interacting protein
KASH: Klarsicht/ANC-1/Syne-1 homology
TF: Transcription factor
MAS: Marker-assisted selection
